# Elevated visceral adiposity index is associated with increased stroke prevalence and earlier age at first stroke onset: Based on a national cross-sectional study

**DOI:** 10.3389/fendo.2022.1086936

**Published:** 2023-01-16

**Authors:** Qingjie Chen, Ziwen Zhang, Ning Luo, Yilong Qi

**Affiliations:** Department of Neurosurgery, The 902nd Hospital of The Chinese People’s Liberation Army, Bengbu, China

**Keywords:** stroke prevalence, stroke onset age, VAI, cross-sectional study, metabolic syndrome

## Abstract

**Objective:**

The purpose of this study was to examine the association between the VAI (visceral adiposity index) and stroke prevalence and age at stroke in US adults.

**Methods:**

We examined the association between VAI and stroke prevalence and age at stroke using logistic regression, subgroup analysis, and dose-response curves using participants from the National Health and Nutrition Examination Survey (NHANES) database from 2007-2018.

**Results:**

This study ultimately included 29,337 participants aged >20 years, of whom 1022 self-reported a history of stroke, and after adjusting for all confounders, each unit increase in corrected VAI was associated with a 12% increase in the prevalence of stroke (OR= 1.12, 95% CI: 1.01, 1.24) along with an earlier age at stroke 1.64 years (β= -1.64, 95% CI: -2.84, -0.45), stratified analysis showed that the prevalence of stroke was 20% higher in the female group (OR= 1.20, 95% CI: 1.04, 1.39), black group (OR= 1.22, 95% CI: 1.01, 1.48), age ≤60 years group (OR= 1.25, 95% CI: 1.05, 1.48), hypertensive group (OR=1.15, 95% CI:1.01, 1.31), and diabetic group (OR=1.23, 95% CI:1.02, 1.48) VAI increase was positively correlated with stroke prevalence increase. The dose-response curves showed a positive linear correlation between increased VAI and stroke prevalence, while a negative linear correlation was observed between increased VAI and age at stroke.

**Conclusion:**

Although a causal relationship cannot be proven, higher VAI was positively associated with stroke prevalence and can lead to earlier stroke onset.

## 1 Introduction

Globally, stroke is the third leading cause of death and the leading contributor to persistent and acquired disability in adults. Approximately 70%-80% of strokes are ischemic strokes, with hemorrhagic strokes accounting for the remainder (1, 2). From 2009 to 2012, a survey of adults aged 20 years or older showed that the overall prevalence was about 2.6% (3). Strokes occur in about 17.8% of people over 45 years old, and asymptomatic cerebral infarction occurs in 6%-28% of those over 45 years old (4). Strokes cost the country and individuals an estimated $45.5 billion each year in 2014-2015 (5), which is a serious economic burden. Public health must take stroke prevention seriously because stroke is a major public health issue.

At present, there is evidence that metabolic syndrome is a combination of risk factors for stroke development, including atherosclerotic dyslipidemia, hypertension, insulin resistance, and obesity, all of which contribute to atherosclerotic vascular disease (6). There is a high risk of stroke and recurrent stroke for people with metabolic syndrome, according to several studies (7–9). With obesity on the rise, the prevalence of metabolic syndrome is expected to rise substantially as obesity increases in the future (10). In turn, this increase in stroke prevalence may place a heavier burden on society due to the close association between metabolic syndrome and obesity. There are limited reliable indicators of obesity that can be used to predict and assess stroke risk despite obesity being strongly associated with stroke.

Adipocytes store triglycerides in adipose tissue, which controls lipid metabolism and glucose homeostasis (11). In addition to storing energy, adipose tissue performs an active endocrine function. Numerous bioactive substances are produced in the body by fat cells, lipid-resident immune cells, and endothelial cells (12). Diabetes, hypertension, cardiovascular disease, and cardiometabolic risk factors are more closely linked to visceral adipose tissue than subcutaneous adipose tissue (13–15). Body fat can be assessed with a variety of methods, including densitometry (dual-energy X-ray absorptiometry, DXA), magnetic resonance imaging (MRI), computed tomography (CT), and mechanical methods. These methods have a high degree of accuracy in assessing body fat, and the first three methods also provide fat imaging and location within the body (16). The costs and time involved in these procedures make them unsuitable for routine use in clinical practice because they are technically complex and expensive.

An adipose tissue function indicator, the visceral adiposity index (VAI) measures the distribution of abdominal fat. Based on waist circumference (WC), body mass index (BMI), triglycerides (TG), and high density lipoprotein (HDL) cholesterol, it is a novel and specific index that indirectly measures visceral adipose function (17). Compared with traditional parameters such as waist circumference and body mass index, the VAI is said to be more sensitive and specific. There has been some progress made in the use of VAI in cardiovascular disease risk assessment associated with obesity (17–19). While it has been reported that VAI is associated with stroke, a study by Zhang et al. (20) found that VAI was positively associated with angina pectoris, heart attack, stroke, hypertension, and coronary artery disease, and a study by Cui et al. (21) found an association between VAI and sudden stroke in Chinese people. However, fewer covariates were included in Zhang and Cui’s study, and more covariates need to be included to assess the relationship. Furthermore, VAI and stroke onset age were included in the study, which has not yet been published. As a result, in this study, we set out to determine if VAI was useful in predicting stroke onset and stroke age in the US adult population.

## 2 Materials and methods

### 2.1 Study population

**Figure 1:**
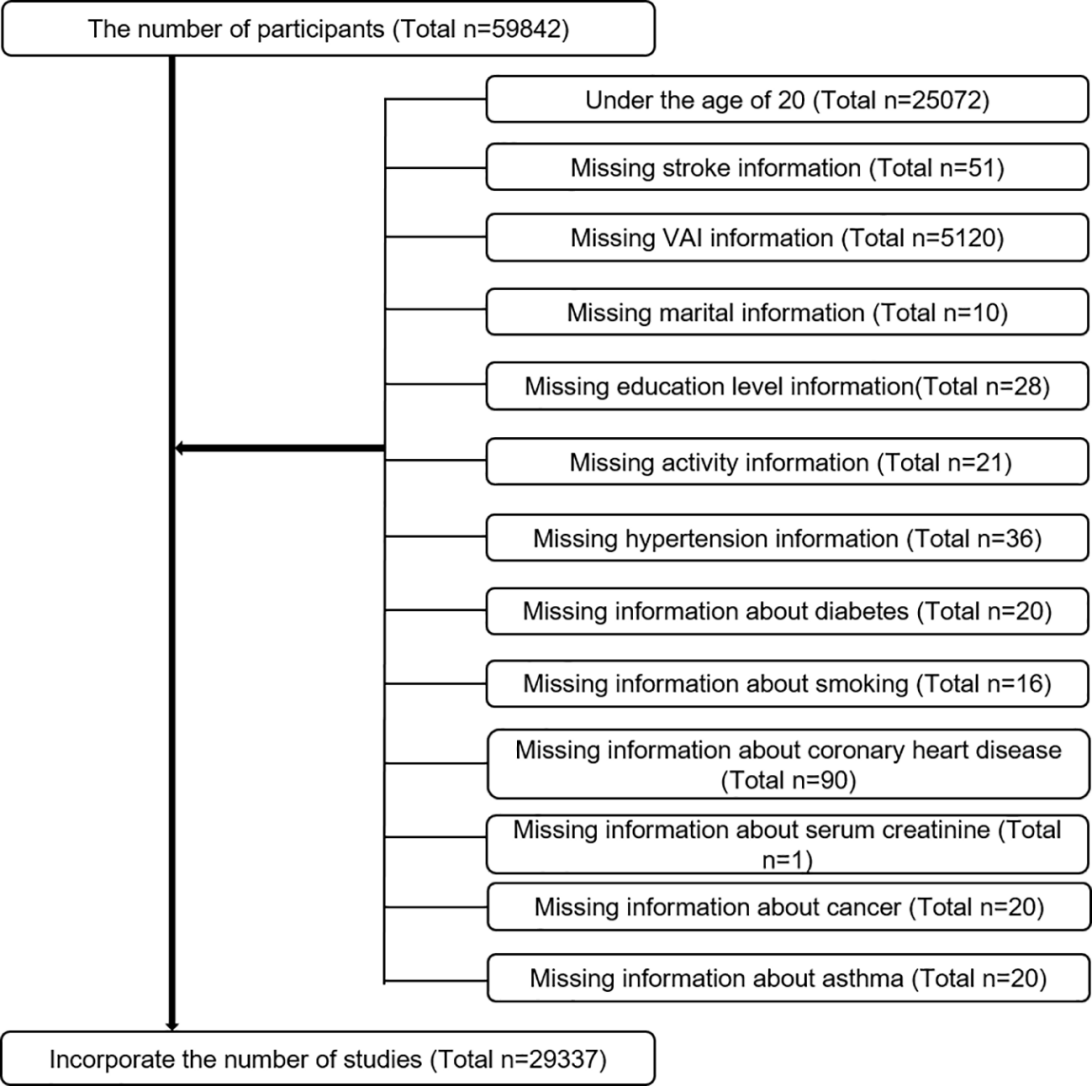
Sample selection process flow chart.

Based on National Health and Nutrition Examination Survey (NHANES) data collected between 2007 and 2018, we evaluated baseline clinical data from only participants over 20 years old who completed the stroke questionnaire, and we analyzed data on participants who explicitly responded to whether they had suffered a stroke. The questionnaire was completed by 59842 people. Exclusion criteria were as follows (**Figure 1**). Finally, 29337 cases, including 1022 self-reported stroke cases, were included in this study.

### 2.2 Data collection and definition

As an exposure variable, VAI was developed. The following sex-specific equations were used to calculate VAI, where the units for WC, BMI, and TG and HDL are cm kg/m2, and mmol/L (22). A biochemical analysis used an enzyme-based method to determine triglyceride concentrations. With the Roche Cobas 6000 chemistry analyzer and Roche Modular P chemistry analyzer, serum triglyceride concentrations were measured. Stroke presence or absence and age at stroke onset were assessed by questionnaires. The presence or absence of stroke and the age at stroke were designed as outcome variables.

Multivariate adjusted models summarized potential covariates that may confound the association between VAI and stroke. Covariates in our study included sex (male/female), age (years), race, education level, poverty to income ratio (PIR), marital status (married or living with partner/single), alcohol consumption (drinking or not), physical activity (vigorous/moderate/below moderate), cholesterol level (mg/dl), fasting glucose (mg/dl), urine protein creatinine ratio (mg/g), smoking status (smoking or not), hypertension (smoking or not), diabetes (smoking or not), coronary heart disease (smoking or not), cancers (yes or not), and dietary intake factors, including energy intake, fat intake, sugar intake, and water intake. All participants in years 2007-2018 with two 24-hour dietary recalls will have their consumption averaged based on the two recalls. The numerical variables with more missing data were converted to categorical variables, and the lowest dichotomous was used as the benchmark. The CDC has posted all detailed measurements of the study variables online at www.cdc.gov/nchs/nhanes/. All NHANES protocols were implemented in accordance with the U.S. Department of Health and Human Services (HHS) Human Research Subject Protection Policy and were reviewed and standardized annually by the NCHS Research Ethics Review Committee. All subjects who participated in the survey signed an informed consent form. All data in this study were released free of charge by NHANES without additional authorization or ethical review.

Smoking status (SMQ020 - Smoked at least 100 cigarettes in life), diabetes (DIQ010 - Doctor told you have diabetes), coronary heart disease (MCQ160C - Ever told you had coronary heart disease), and cancer (MCQ220 - Ever told you had cancer or malignancy) were obtained from the questionnaire data. Participants were considered to have the disease when they answered “yes”. For hypertension using blood pressure monitoring data from the physical examination, NHANES obtained three consecutive blood pressure readings after participants rested quietly in a seated position for 5 minutes and determined the maximum inflation level (MIL), we took the average of the three tests and converted them into categorical variables according to 140/90 mmHg, with missing values forming their own dummy variable group. The activity data was obtained from the activity questionnaire(PAQ605 - Vigorous work activity, PAQ620 - Moderate work activity, PAQ650 - Vigorous recreational activities, PAQ665 - Moderate recreational activities), when there was strenuous work or recreational activity was identified as the strenuous activity group, when there was moderate work or recreational activity was identified as the moderate activity group, and when there was none of the above activities was considered as the inactive group.

When continuous variables have a large number of missing values, we convert them to categorical variables (23, 24), where the missing values form their own group as a dummy variable group.

### 2.3 Statistical methods

To demonstrate the complex, multi-stage sampling design used in selecting a representative U.S. non-institutionalized population, all statistical analyses were conducted using the sampling weights, stratification, and clustering provided in the NHANES study. A weighted survey mean and 95% confidence intervals are used to express continuous variables, and a weighted survey mean and 95% confidence intervals are used to express categorical variables. Due to the skewed distributions of VAI, LN transformations are applied to transform them into normal distributions. All covariates were screened for variance inflation factor (VIF) covariance, and if the VIF value exceeded 5, the covariate was removed. As per the guidelines, multiple logistic regression models were used to explore the VAI, different VAI triplet groups, and stroke prevalence in three different models based on the Strengthening the Reporting of Observational Studies in Epidemiology (STROBE) statement (25). As far as model 1 is concerned, no adjustment for covariates was made. Several factors were adjusted in model 2, including gender, age, race, marital status, and education. Adjustments were made to all variables in model 3. To further clarify the relationship between VAI and stroke, we used a propensity score method and performed sensitivity analyses. Smoothed curve fitting (penalized spline method) and generalized additive model regression (GAM) were carried out. When a nonlinear relationship was determined to exist, likelihood ratio tests were used to determine inflection point values. Multiple regression analyses were next performed stratified by sex, age, race, hypertension, and diabetes. p < 0.05 was considered statistically significant. All analyses were performed using Empower software www.empowerstats.com (X&Y Solutions, Inc., Boston, Massachusetts, USA) and R version 4.0.2 (http://www.R-project.org, The R Foundation).

## 3 Results

The demographic characteristics of the included participants are shown in **Table 1**. The VAI was 0.72 (0.66,0.78) in the stroke group, higher than 0.56 (0.54,0.58) in the normal group, p < 0.0001. There was a significant difference between the stroke group and the normal group in the proportion of blacks, age, prevalence of hypertension, and prevalence of diabetes.

**Table 1:** Baseline characteristics of participants, weighted.

Characteristic	Non-stroke formers (*n* = 28315)	Stroke formers (*n* = 1022)	P-value
Age(years)	46.78 (46.34,47.23)	63.53 (62.32,64.74)	<0.0001
Serum Cholesterol (mg/dl)	194.20 (193.19,195.22)	184.53 (180.65,188.41)	<0.0001
Ln(VAI)	0.56 (0.54,0.58)	0.72 (0.66,0.78)	<0.0001
Serum Creatinine(mg/dl)	0.87 (0.87,0.88)	1.05 (1.01,1.10)	<0.0001
Serum Glucose(mg/dl)	99.12 (98.56,99.67)	112.35 (109.10,115.61)	<0.0001
Gender(%)			0.0133
Male	48.66 (48.06,49.26)	43.21 (38.98,47.55)	
Female	51.34 (50.74,51.94)	56.79 (52.45,61.02)	
Race(%)			<0.0001
Mexican American	14.82 (12.92,16.94)	7.83 (6.21,9.83)	
White	66.66 (63.79,69.41)	69.36 (64.97,73.42)	
Black	10.49 (9.19,11.94)	15.48 (12.88,18.49)	
Other Race	8.03 (7.19,8.96)	7.33 (5.31,10.05)	
Education Level(%)			<0.0001
Less than high school	20.02 (18.65,21.46)	34.03 (30.25,38.02)	
High school	28.89 (27.76,30.05)	30.46 (26.63,34.58)	
More than high school	51.09 (49.22,52.96)	35.52 (31.08,40.22)	
Marital Status(%)			0.0199
Cohabitation	64.14 (62.88,65.39)	58.83 (54.15,63.35)	
Solitude	35.86 (34.61,37.12)	41.17 (36.65,45.85)	
Alcohol(%)			0.0009
Yes	61.23 (59.76,62.68)	53.91 (49.87,57.89)	
No	18.47 (17.39,19.60)	23.41 (19.97,27.24)	
Unclear	20.30 (19.21,21.43)	22.69 (19.39,26.36)	
Diabetes(%)			<0.0001
Yes	8.87 (8.41,9.35)	29.42 (26.14,32.93)	
No	91.13 (90.65,91.59)	70.58 (67.07,73.86)	
Smoked(%)			<0.0001
Yes	43.77 (42.56,44.99)	59.92 (56.02,63.69)	
No	56.23 (55.01,57.44)	40.08 (36.31,43.98)	
Physical Activity(%)			<0.0001
Never	25.77 (24.83,26.75)	48.58 (44.45,52.73)	
Moderate	31.90 (30.99,32.82)	32.45 (28.65,36.50)	
Vigorous	42.33 (41.24,43.42)	18.96 (15.84,22.54)	
Asthma(%)			<0.0001
No	85.52 (84.85,86.17)	75.82 (72.52,78.85)	
Yes	14.48 (13.83,15.15)	24.18 (21.15,27.48)	
Coronary Artery Disease			<0.0001
Yes	2.98 (2.64,3.35)	19.20 (15.86,23.05)	
No	97.02 (96.65,97.36)	80.80 (76.95,84.14)	
Cancers			<0.0001
Yes	9.63 (9.17,10.10)	23.08 (19.11,27.59)	
No	90.37 (89.90,90.83)	76.92 (72.41,80.89)	
Ratio of Family Income to Poverty			<0.0001
<1.3	19.66 (18.48,20.90)	31.17 (27.34,35.27)	
≥1.3<3.5	32.60 (31.35,33.87)	39.59 (35.19,44.17)	
≥3.5	40.45 (38.59,42.33)	22.31 (18.49,26.65)	
Unclear	7.29 (6.67,7.96)	6.93 (5.27,9.07)	
Total Kcal(%)			<0.0001
Lower	38.86 (38.07,39.65)	52.72 (48.30,57.10)	
Higher	46.40 (45.47,47.33)	33.97 (30.25,37.89)	
Unclear	14.75 (13.94,15.59)	13.31 (10.59,16.61)	
Total Sugar(%)			0.0444
Lower	36.40 (35.58,37.22)	40.86 (37.04,44.79)	
Higher	37.22 (36.31,38.13)	36.63 (32.36,41.12)	
Unclear	26.38 (25.61,27.17)	22.51 (19.25,26.15)	
Total Fat(%)			<0.0001
Lower	38.42 (37.49,39.35)	50.85 (47.00,54.70)	
Higher	46.83 (45.89,47.78)	35.83 (32.50,39.31)	
Unclear	14.75 (13.94,15.59)	13.31 (10.59,16.61)	
High Blood Pressure(%)			<0.0001
No	78.26 (77.24,79.24)	62.32 (57.94,66.51)	
Yes	12.70 (12.09,13.33)	24.13 (21.04,27.52)	
Unclear	9.04 (8.23,9.94)	13.55 (10.34,17.55)	
Urine Albumin Creatinine Ratio(%)			<0.0001
Lower	54.86 (53.90,55.82)	32.04 (27.12,37.40)	
Higher	44.59 (43.64,45.54)	65.28 (60.10,70.12)	
Unclear	0.55 (0.46,0.67)	2.68 (1.87,3.83)	
Total Water(%)			<0.0001
Lower	38.42 (37.49,39.35)	50.85 (47.00,54.70)	
Higher	46.83 (45.89,47.78)	35.83 (32.50,39.31)	
Unclear	14.75 (13.94,15.59)	13.31 (10.59,16.61)	

Data of continuous variables are shown as survey-weighted mean(95%CI), P value was calculated by survey-weighted linear regression.Data of categorical variables are shown as survey-weighted percentage (95%CI), P value was calculated by survey-weighted Chi-square test.

### 3.1 A higher VAI is associated with a higher prevalence of stroke

In the final adjusted model, all variables were included if the VIF for each indice was below 5. For stroke, a positive correlation was observed between the VAI and stroke prevalence. Based on the fully adjusted model (model 3), we found a positive association of 1.12 (95% confidence interval: 1.01, 1.21) between the LN-transformed VAI and stroke prevalence of 12%. Furthermore, in order to analyze sensitivity, the VAI was transformed into a categorical variable (dichotomous). Increased prevalence of stroke in high dichotomies compared to the lowest VAI dichotomous group, but there was no effect value (OR=1.10, 95% CI: 0.95, 1.28). (**Table 2**). Each unit increase in VAI value after propensity matching was associated with a 29% increase in adjusted stroke prevalence (OR=1.29,95% CI:1.14,1.47) (**Supplementary Tables 1**, **2**).

**Table 2:** Analysis between VAI with stroke prevalence.

Characteristic	Model 1 OR(95%CI)	Model 2 OR(95%CI)	Model 3 OR(95%CI)
Ln(VAI)	1.20 (1.11, 1.29)	1.22 (1.12, 1.33)	1.12 (1.01, 1.24)
Categories			
Lower	1	1	1
Higher	1.30 (1.14, 1.47)	1.25 (1.10, 1.43)	1.10 (0.95, 1.28)

Model 1=no covariates were adjusted.

Model 2=Model 1+age, gender, race education, marital status were adjusted.

Model 3=Model 2+, diabetes, blood pressure, asthma, PIR, total water, total kcal, total sugar, smoked, physical activity, alcohol use, serum cholesterol, coronary artery disease, serum creatinine urine albumin creatinine ratio, cancers and serum glucose were adjusted.

### 3.2 Analysis of the dose-response and threshold effects of VAI on stroke prevalence

**Figure 2:**
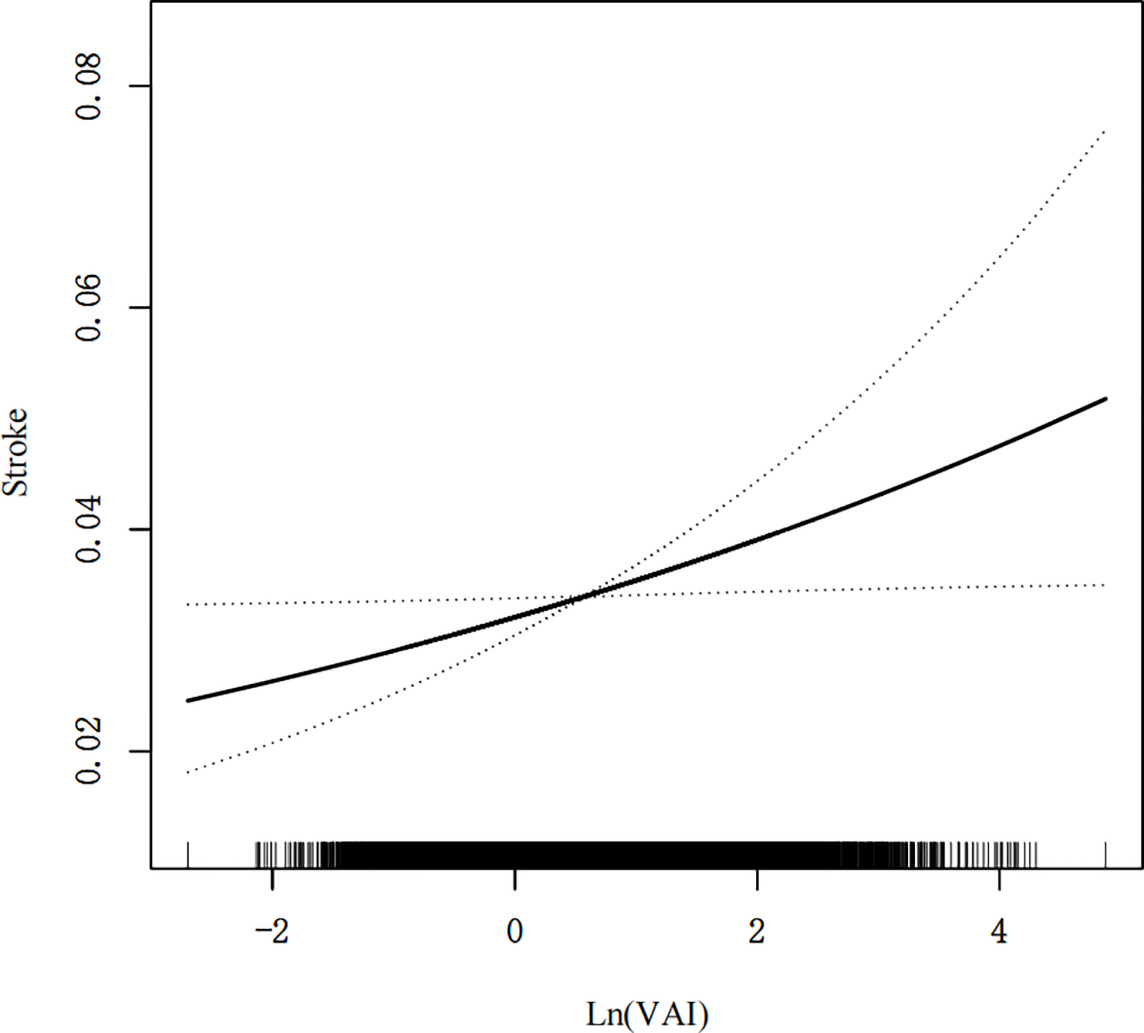
Density dose-response relationship between VAI with stroke prevalence. The area between the upper and lower dashed lines is represented as 95% CI. Each point shows the magnitude of the VAI and is connected to form a continuous line. Adjusted for all covariates except effect modifier.

A generalized additive model and smoothed curve fitting were used to investigate the relationship between the VAI and stroke prevalence. According to our findings (**Figure 2**), stroke prevalence was linearly related to the VAI.

#### 3.2.1 Subgroup analysis

Subgroup analyses were performed to assess the robustness of the association between VAI and stroke prevalence. The results showed that in the subgroup analysis the VAI indices in the female group (OR=1.15, 95% CI: 1.01, 1.24), black group (OR=1.22, 95% CI:1.01, 1.48), age ≤60 years group (OR=1.25, 95% CI:1.05, 1.48), hypertension group (OR=1.15, 95% CI:1.01, 1.31), and diabetes group (OR=1.23, 95% CI:1.02, 1.48) VAI increase were all positively associated with increased prevalence of stroke. (**Table 3**).

**Table 3:** Subgroup analysis between VAI with stroke prevalence.

Characteristic	Model 1 OR(95%CI)	Model 2 OR(95%CI)	Model 3 OR(95%CI)
Stratified by gender			
Male	1.01 (0.91, 1.13)	1.13 (1.00, 1.27)	1.06 (0.92, 1.22)
Female	1.43 (1.29, 1.60)	1.35 (1.20, 1.53)	1.20 (1.04, 1.39)
Stratified by race			
Mexican American	1.10 (0.90, 1.34)	1.01 (0.82, 1.26)	0.98 (0.75, 1.28)
White	1.36 (1.22, 1.52)	1.30 (1.15, 1.46)	1.14 (0.99, 1.32)
Black	1.37 (1.18, 1.59)	1.29 (1.10, 1.52)	1.22 (1.01, 1.48)
Other Race	1.04 (0.79, 1.38)	0.92 (0.68, 1.25)	0.67 (0.44, 1.01)
Stratified by age			
<60	1.39 (1.22, 1.58)	1.52 (1.33, 1.74)	1.25 (1.05, 1.48)
≥60	1.04 (0.94, 1.15)	1.10 (0.99, 1.22)	1.01 (0.89, 1.14)
Stratified by hypertension			
YES	1.28 (1.16, 1.41)	1.25 (1.12, 1.39)	1.15 (1.01, 1.31)
NO	0.95 (0.82, 1.09)	1.11 (0.95, 1.30)	1.02 (0.84, 1.22)
Stratified by diabetes			
YES	1.07 (0.93, 1.23)	1.19 (1.02, 1.39)	1.23 (1.02, 1.48)
NO	1.08 (0.98, 1.18)	1.10 (1.00, 1.22)	1.08 (0.95, 1.21)

Model 1=no covariates were adjusted.

Model 2=Model 1+age, gender, race education, marital status were adjusted.

Model 3=adjusted for all covariates except effect modifier.

### 3.3 Elevated VAI and earlier age of stroke onset

Using the fully adjusted model 3, for every one unit increase in Ln (VAI), stroke onset age was 1.64 years earlier (OR=-1.64, 95% CI: -2.84, -0.45) (**Table 4**).

**Table 4:** Analysis between VAI with stroke age onset.

Characteristic	Model 1 β(95%CI)	Model 2 β(95%CI)	Model 3 β(95%CI)
Ln(VAI)	-0.79 (-1.92, 0.35)	-1.34 (-2.51, -0.18)	-1.64 (-2.84, -0.45)

Model 1=no covariates were adjusted.

Model 2=Model 1+gender, race education, marital status were adjusted.

Model 3=Model 2+, diabetes, blood pressure, asthma, PIR, total water, total kcal, total sugar, smoked, physical activity, alcohol use, serum cholesterol, coronary artery disease, serum creatinine urine albumin creatinine ratio, cancers and serum glucose were adjusted.

### 3.4 Analysis of the dose response and threshold effect of VAI on age at stroke onset

**Figure 3:**
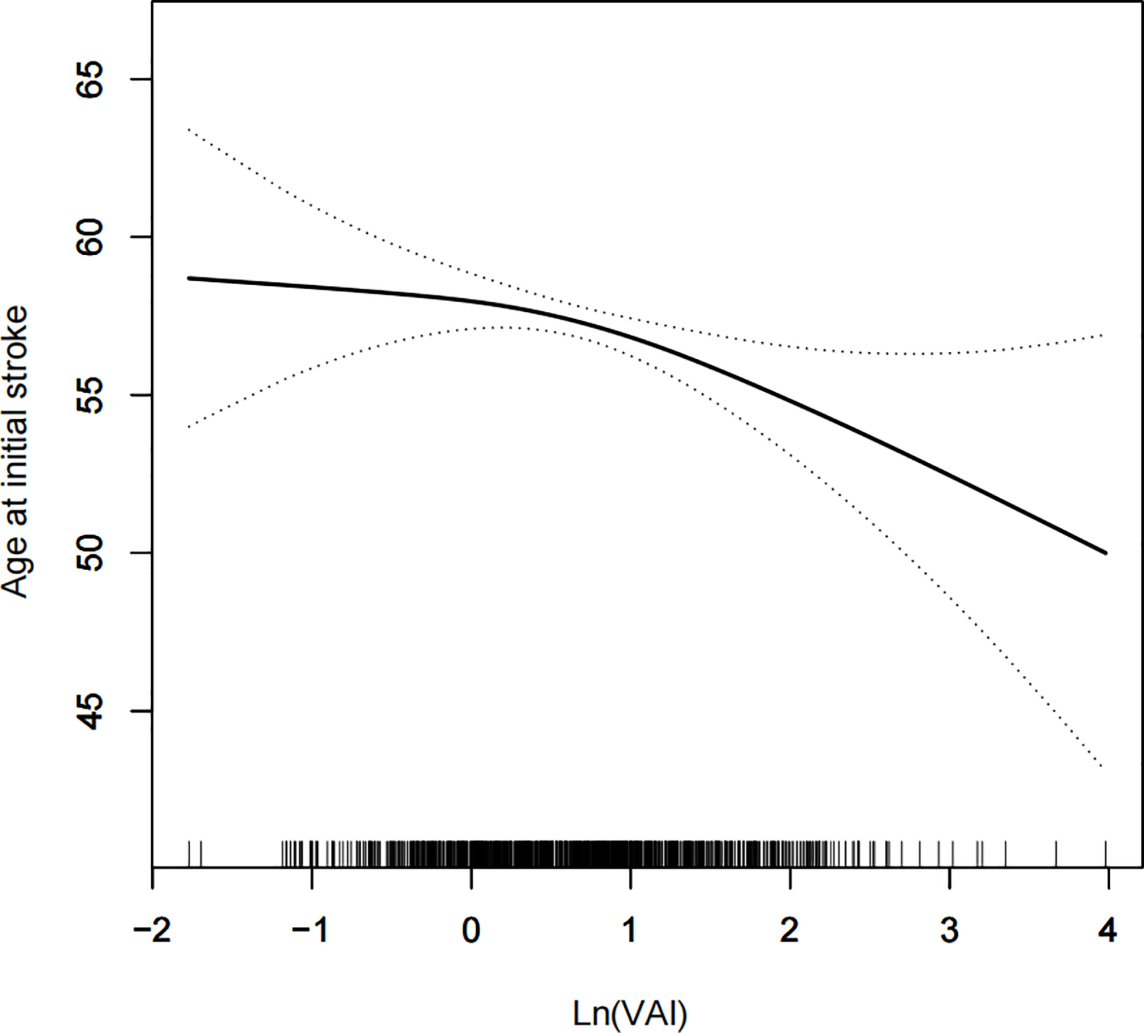
Density dose-response relationship between VAI with onset age of stroke. The area between the upper and lower dashed lines is represented as 95% CI. Each point shows the magnitude of the VAI and is connected to form a continuous line. Adjusted for all covariates except effect modifier.

A generalized additive model and smoothed curve fitting were used to examine the relationship between the VAI and age at stroke onset. Based on our results, VAI increased with increasing age at stroke onset in a negative linear relationship (**Figure 3**).

## 4 Discussion

As a result of industrialization and urbanization, there has been an increase in the consumption of food and lifestyle, suggesting that these changes are risk factors for stroke development (26, 27) . Thus, epidemiological studies of stroke onset associated with metabolic syndrome are reasonable. As well, VAI is more sensitive and specific than traditional waist circumference and BMI for obesity (18, 19, 28) . Therefore, in this study, we investigated the relationship between VAI levels and stroke in a large U.S. population and found that after adjusting for all confounders, increased VAI levels were positively correlated with stroke prevalence, and age at stroke was negatively correlated with increased VAI levels. The age of first stroke onset was 1.64 years earlier with each unit increase in VAI after correction, and the prevalence of stroke increased 12% after correction.

Stroke affects both physical and mental health severely, placing an immense burden on our society in terms of morbidity, quality of life, and healthcare costs. These pressures continue to rise throughout the world, making stroke prevention particularly crucial. The VAI can also be used to find specific populations adapted to the index and prevent strokes from occurring more often. Consequently, we performed subgroup analyses on females, blacks, those aged >60, hypertensives, and diabetics and found elevated VAI levels were positively associated with increased stroke prevalence. According to several previous related studies, we suspect this finding to be accurate. As previously reported, VAI differences have been found in correlation studies of atherosclerosis, cardiovascular disease, and asymptomatic cerebral infarction (17, 21, 29–31) . A study by Li et al (32) showed that women with VAI were associated with intracranial atherosclerosis, but not men. The findings of Nakagomi (33) also indicate that VAI increases atherosclerosis in women and the association is stronger. As well as predicting cardiometabolic disease in older women, VAI has been found useful in cardiovascular disease studies - a score of 2.71 can be used to identify high-risk women (34). Based on the same study by Mohammadreza (35), women were independently at an increased risk of cardiovascular disease after VAI. According to a Korean study published in 2020, high VAI levels were associated with an increased risk of asymptomatic cerebral infarction in healthy females, particularly (36). Nakagomi (33) speculates that the possible explanation for the above findings is either differences in hormone levels between men and women or differences in the composition of visceral adipose tissue and subcutaneous adipose tissue. However, the etiologic mechanism behind these findings remains unclear. As a result of our study, VAI has a greater effect on stroke risk among younger people than those older than 60 years of age. So far, it seems that young and middle-aged individuals have different risk factors for stroke compared to elderly individuals. Atherosclerosis (including atrial fibrillation), hypertension and diabetes mellitus are the three most common risk factors in the elderly (37). Among young and middle-aged stroke patients, dyslipidemia, smoking and hypertension are the most prevalent vascular risk factors (38–40), while VAI levels are also affected by dyslipidemia, which makes it possible to predict stroke prevalence in young and middle-aged individuals. It is well known that stroke risk differs by race and ethnicity, and in younger populations these differences are even more pronounced. It is important to note that variations in prevalence are largely determined by the racial composition of the study population. There was an increased prevalence of strokes among young Hispanics and blacks in the Northern Manhattan study (41). The hospitalization rate for stroke was significantly higher among young blacks and Hispanics in another Florida study (42). A second study from American Point found that young and middle-aged blacks had up to five times the stroke risk, compared to young and middle-aged whites. Blacks had an increased prevalence of stroke due to the elevated VAI found in our study, which may explain the increased prevalence of stroke among blacks. The increased prevalence of stroke in populations with hypertension and diabetes is not surprising given that both of these factors are known to be risk factors for stroke (43).

In terms of mortality, stroke is one of the top three causes of death worldwide, as well as one of the leading causes of disability. If stroke continues for a longer period of time, the risk of a second stroke increases, as does the poorer prognosis. The long-term socioeconomic consequences of stroke in young patients are also significant. According to a recent study, young stroke patients spend an average of $34,886 in hospitalization for ischemic stroke, $150,307 for subarachnoid hemorrhage, and $94,482 for cerebral hemorrhage (44). The correlation between VAI and age at first stroke was another important finding in this study. As a consequence of our results, every unit increase in VAI will result in a 1.64 year increase in the age of stroke onset. VAI was linearly correlated negatively with age at first stroke even when smoothing curves were fitted. This finding is heartening and has not yet been reported. It is hypothesized that treating and managing VAI levels at younger ages can reduce the risk of stroke. However, the veracity of the present results may be limited by the sample size and needs to be further confirmed by a multicenter prospective study with a large sample.

Several advantages are associated with our study. An extensive quality assurance and quality control process is followed by the NHANES 2007-2018 sample, which represents a representative sample of the U.S. population. Secondly, we adjusted for confounding covariates to ensure our results were reliable and applicable to a wide range of individuals. Our study does, however, have some limitations. We were unable to establish a causal relationship between VAI and stroke due to the fact that we used the NHANES database, a cross-sectional study. In addition, the data on medication history and stroke type classification were not disclosed in the database, which may have contributed to recall bias. Third, the diagnosis of stroke was made by means of a questionnaire, which can be subject to recall bias. It is noteworthy that the present study showed that VAI is associated with stroke onset and, for the first time, evaluated its role in age at first stroke onset.

## 5 Summary

The VAI is associated with higher stroke prevalence and a younger age at first stroke. Although the causal relationship between VAI management and stroke occurrence cannot be clearly established, we hypothesize that managing VAI levels at a younger age may reduce the occurrence of strokes and delay stroke onset.

## Data availability statement

The original contributions presented in the study are included in the article/**Supplementary Materials**, further inquiries can be directed to the corresponding author.

## Ethics statement

The studies involving human participants were reviewed and approved by NCHS Research Ethics Review Committee. The patients/participants provided their written informed consent to participate in this study.

## Author contributions

QC: Conceptualization, Methodology, Software. ZZ: Data curation, Writing original draft. NL: Visualization, Investigation. YQ: Writing - review & editing. All authors contributed to the article and approved the submitted version.
