# Supplementary material for: Elevated visceral adiposity index is associated with increased stroke prevalence and earlier age at first stroke onset: Based on a national cross-sectional study
Source: Front Endocrinol (Lausanne). 2023 Jan 16;13:1086936. doi: 10.3389/fendo.2022.1086936 (PMC9884813; doi:10.3389/fendo.2022.1086936)
Supplement: Supplementary file 1 [file Table_1.docx]

Supplementary Table 1.Baseline information after propensity score matching

| Variables | Non-stroke formers | **Stroke formers** | Standardized diff. | P value |
| --- | --- | --- | --- | --- |
| Age(years) | (1022) 42.19 ±21.57 | (1022) 64.97 ± 13.17 | 1.2751 | <0.0001 |
| Ln(VAI ) | (1022) 0.52 ± 0.76 | (1022) 0.69 ±0.79 | 0.2263 | <0.0001 |
| Gender(%) |  |  | 0.0862 | 0.0571 |
| Male | 530 (51.9) | 486 (47.6) |  |  |
| Female | 492 (48.1) | 536 (52.4) |  |  |
| Race(%) |  |  |  | <0.0001 |
| Mexican American | 517 (50.6) | 158 (15.5) | 0.8052 |  |
| White | 375 (36.7) | 500 (48.9) | 0.2491 |  |
| Black | 83 (8.1) | 291 (28.5) | 0.5456 |  |
| Other Race | 47 (4.6) | 73 (7.1) | 0.1084 |  |
| Education Level(%) |  |  |  | 0.0113 |
| Less than high school | 349 (34.1) | 404 (39.5) | 0.1117 |  |
| High school | 357 (34.9) | 300 (29.4) | 0.1196 |  |
| More than high school | 316 (30.9) | 318 (31.1) | 0.0042 |  |
| Marital Status(%) |  |  | 0.1019 | 0.0241 |
| [Cohabitation](#keyfrom=E2Ctranslation) | 484 (47.4) | 536 (52.4) |  |  |
| [Solitude](#keyfrom=E2Ctranslation) | 538 (52.6) | 486 (47.6) |  |  |
| Alcohol(%) |  |  |  | <0.0001 |
| Yes | 521 (51) | 520 (50.9) | 0.002 |  |
| No | 190 (18.6) | 260 (25.4) | 0.1659 |  |
| Unclear | 311 (30.4) | 242 (23.7) | 0.1524 |  |
| Diabetes(%) |  |  | 0.581 | <0.0001 |
| Yes | 98 (9.6) | 330 (32.3) |  |  |
| No | 924 (90.4) | 692 (67.7) |  |  |
| Smoked(%) |  |  | 0.4853 | <0.0001 |
| Yes | 386 (37.8) | 627 (61.4) |  |  |
| No | 636 (62.2) | 395 (38.6) |  |  |
| Physical Activity(%) |  |  |  | <0.0001 |
| Never | 289 (28.3) | 525 (51.4) | 0.4854 |  |
| Moderate | 300 (29.4) | 320 (31.3) | 0.0426 |  |
| Vigorous | 433 (42.4) | 177 (17.3) | 0.5692 |  |
| Asthma(%) |  |  | 0.1422 | 0.0016 |
| No | 855 (83.7) | 798 (78.1) |  |  |
| Yes | 167 (16.3) | 224 (21.9) |  |  |
| Coronary Artery Disease |  |  | 0.5384 | <0.0001 |
| Yes | 25 (2.4) | 187 (18.3) |  |  |
| No | 997 (97.6) | 835 (81.7) |  |  |
| Cancers |  |  | 0.4133 | <0.0001 |
| Yes | 78 (7.6) | 225 (22) |  |  |
| No | 944 (92.4) | 797 (78) |  |  |
| PIR |  |  |  | <0.0001 |
| ＜1.3 | 335 (32.8) | 391 (38.3) | 0.1147 |  |
| ≥1.3＜3.5 | 341 (33.4) | 390 (38.2) | 0.1002 |  |
| ≥3.5 | 235 (23) | 169 (16.5) | 0.1627 |  |
| Unclear | 111 (10.9) | 72 (7) | 0.134 |  |
| Total Kcal(%) |  |  |  | <0.0001 |
| Lower | 418 (40.9) | 551 (53.9) | 0.2629 |  |
| Higher | 419 (41) | 317 (31) | 0.209 |  |
| Unclear | 185 (18.1) | 154 (15.1) | 0.0816 |  |
| Total Sugar(%) |  |  |  | 0.3689 |
| Lower | 385 (37.7) | 415 (40.6) | 0.0602 |  |
| Higher | 371 (36.3) | 360 (35.2) | 0.0225 |  |
| Unclear | 266 (26) | 247 (24.2) | 0.0429 |  |
| Total Water(%) |  |  |  | 0.0001 |
| Lower | 427 (41.8) | 522 (51.1) | 0.1872 |  |
| Higher | 410 (40.1) | 346 (33.9) | 0.13 |  |
| Unclear | 185 (18.1) | 154 (15.1) | 0.0816 |  |
| Total Fat(%) |  |  |  | 0.0001 |
| Lower | 427 (41.8) | 522 (51.1) | 0.1872 |  |
| Higher | 410 (40.1) | 346 (33.9) | 0.13 |  |
| Unclear | 185 (18.1) | 154 (15.1) | 0.0816 |  |
| High Blood Pressure(%) |  |  |  | <0.0001 |
| No | 811 (79.4) | 600 (58.7) | 0.4581 |  |
| Yes | 119 (11.6) | 300 (29.4) | 0.4497 |  |
| Unclear | 92 (9) | 122 (11.9) | 0.096 |  |
| Urine Albumin Creatinine Ratio(%) |  |  |  | <0.0001 |
| Lower | 531 (52) | 297 (29.1) | 0.4796 |  |
| Higher | 485 (47.5) | 693 (67.8) | 0.4209 |  |
| Unclear | 6 (0.6) | 32 (3.1) | 0.1892 |  |

For continuous variables: (N) Mean ± SD, Standardized difference = abs(Mean1-Mean0)/sqrt((S1+S2)/2)

For categorical variables: N (%), Standardized difference = abs(P1-P0)/sqrt((P1*(1-P1)+P0*(1-P0))/2)

Supplementary Table 2

| **Characteristic** | **Model** 1 OR(95%CI) | **Model** 2 OR(95%CI) | **Model** 3 OR(95%CI) |
| --- | --- | --- | --- |
| Ln(VAI ) | 1.34 (1.20, 1.50) | 1.38 (1.20, 1.59) | 1.29 (1.14, 1.47) |

Model 1=no covariates were adjusted.

Model 2=Model 1+age, gender, race education, marital status were adjusted.

Model3=Model 2+,diabetes,blood pressure, asthma, PIR, total water, total kcal, total sugar, smoked, physical activity, alcohol use, serum cholesterol, coronary artery disease, serum [creatinine](http://www.baidu.com/link?url=z0JPzMXFfp9dv8u03FXVF9QrZIiZhFV-5qIc-uCLoQkpg-xAGzIXXHqjW7E2CRh6DkH3MZJ4lKNot5mFpj0yHCemz1u3TqnZubiUFbRjNT7) urine albumin creatinine ratio, cancers and serum glucose were adjusted.
